# *Pseudomonas* sp. N5.12 Metabolites Formulated in AgNPs Enhance Plant Fitness and Metabolism Without Altering Soil Microbial Communities

**DOI:** 10.3390/plants14111655

**Published:** 2025-05-29

**Authors:** Svitlana Plokhovska, Ana García-Villaraco, Jose Antonio Lucas, Francisco Javier Gutiérrez-Mañero, Beatriz Ramos-Solano

**Affiliations:** 1 Faculty of Pharmacy, Universidad San Pablo-CEU Universities, 28668 Madrid, Spain; anabec.fcex@ceu.es (A.G.-V.); alucgar@ceu.es (J.A.L.); jgutierrez.fcex@ceu.es (F.J.G.-M.); 2 Institute of Food Biotechnology and Genomics NAS of Ukraine, 04123 Kyiv, Ukraine

**Keywords:** silver nanoparticles, toxicity, cytoskeleton, oxidative stress, microbial communities, environmental safety

## Abstract

This study investigated the effects of metabolites from the beneficial bacteria *Pseudomonas* N5.12 formulated as silver nanoparticles (AgNPs) on tomato plants and soil microbial communities to explore the environmental safety of AgNPs for future applications in agriculture. AgNPs coated with bacterial metabolites exhibit biological activity that is dose-dependent, as shown by cytoskeleton alterations in Arabidopsis roots. The results show that N5.12-AgNPs can trigger beneficial effects on tomato plants, either when delivered through the leaves or roots, indicating the effectiveness of the metabolites formulated as NP. These effects consist of lowering oxidative stress metabolism and, therefore, improving plant resilience and increasing chlorophyll *a* and carotenoid content. The significant reduction in H_2_O_2_ content was not associated with ROS-scavenging enzymes but with an increase in total phenolic content. In contrast, AgNPs had a minimal impact on bacterial metabolic activity, irrespective of the application method. The structure of microbial communities was not altered by AgNPs, indicating environmental safety for agronomic applications. These findings suggest that *Pseudomonas* N5.12 metabolites formulated in AgNPs at physiological concentrations (30 ppm) may offer agricultural benefits by improving plant health and appearing as an environmentally safe alternative for agriculture.

## 1. Introduction

Silver nanoparticles have attracted significant interest due to their physicochemical properties, including their high surface area, ease of functionalization, and antibacterial activity. There are different procedures to reduce Ag^+^ to Ag^0^, from chemical to biological methods, resulting in the synthesis of NPs. Among these, biological synthesis is relevant because it is low-cost and environmentally friendly [1]. The biological synthesis procedure determines the size and shape of the NP, with a unique organic matter coat that stabilizes the NP and modulates its effects. Regardless of their synthesis, NPs are increasingly used in various sectors, including agriculture, medicine, textiles, and electronics [2,3]. As their use in agricultural applications is experiencing great development, from pesticides to fertilizers and plant growth stimulants, it is essential to examine their potential impacts on plant physiology and soil ecosystem safety prior to exploring their implications for food security, as they may also accumulate on edible parts of plants [4].

The interaction of AgNPs with plants has been the subject of extensive research, revealing both negative and positive effects on plant growth and development. After absorption by plant roots, AgNPs can be translocated to the aerial parts, where they influence various physiological processes, including photosynthesis, nutrient uptake, and stress responses [5]. On one hand, AgNPs enhance seed germination and early-stage growth, likely through improved antioxidant activity and hormonal signaling [6]; at low concentrations, AgNPs trigger defense mechanisms and ROS production to help plant acclimation [7], increase nutrient absorption [8], and promote plant growth under different conditions [9,10].

A major concern regarding plant exposure to AgNPs is their ability to trigger oxidative stress in plants, probably due to the release of Ag^+^ ions, which can bind to plant tissues and disrupt cellular functions. At high concentrations, AgNPs can cause toxicity by inducing oxidative stress, damaging plant cell membranes, and disrupting metabolic pathways [11,12]. In line with this, the cytoskeleton is an excellent marker for this goal since it reacts immediately to environmental stress. Both microtubules and microfilaments disassemble upon stress sensing to repolymerize within a short time if the perturbation is not toxic [13]. Hence, alterations in the cytoskeleton structure are a good marker to determine the physiological concentration of AgNPs [14]. Indeed, biologically synthesized AgNPs are usually capped; therefore, oxidation of Ag^0^ is not likely to occur.

Additionally, recent research has highlighted the potential of AgNPs to influence soil microbial functional diversity. AgNPs can alter key microbial processes, such as carbon and nitrogen cycling, leading to changes in soil quality and the availability of essential nutrients for plants [15]. The impact on microbial communities may vary depending on soil characteristics (pH, organic matter content) and the form in which silver is applied. AgNPs are known to exert antimicrobial effects, which can lead to shifts in microbial population dynamics that may be beneficial or detrimental to plants. Numerous studies have shown that nanoparticles (NPs) can enhance plant nutrient uptake and stimulate growth by increasing the diversity of rhizosphere microbiota [15,16]. However, AgNPs can inhibit the growth of soil bacteria, fungi, and other microorganisms, potentially disrupting beneficial microbial communities [17]. Thus, while the use of AgNPs in agriculture holds significant promise, it raises concerns about their potential environmental impact on the structural and functional diversity of microbial communities.

Interestingly, recent studies have reported the synthesis of AgNPs coated with metabolites from two different beneficial *Pseudomonas* strains [18,19]. One of these strains, *Pseudomonas* N5.12, as well as its metabolites, can promote plant growth and trigger the plant’s innate immune system; it also enhances plant growth and yield under water-limiting conditions [20], therefore showing an interesting application for agriculture. However, N5.12-NP was able to inhibit the growth of microorganisms in vitro [18]. Hence, the present study aims to shed light on this apparent contradiction, which is unique to this strain because of its metabolites and will contribute to the general knowledge of NP effects on agronomic soil communities. We hypothesized that bacterial metabolites formulated in AgNP would trigger plant metabolism without altering rhizosphere microbial communities because bacterial metabolites will stabilize the NP on the one hand, and on the other hand, N5.12 is natural to plant’s rhizosphere anticipating a non-aggressive behavior to other microorganisms. As plants determine their own rhizosphere microbial communities through exudates, we explored changes after root application to rule out direct inhibitory effects of AgNPs or leaf delivery to rule out indirect systemic effects through exudates. Therefore, this study aimed to investigate the effects of *Pseudomonas* N5.12 metabolites formulated as AgNPs on plant growth and soil microbial communities. To achieve these goals, a physiological dose will be determined based on cytoskeleton disruption in transgenic *Arabidopsis* plants. Secondly, AgNPs will be delivered to tomato plants through leaves or roots, and their effects on plant physiology (photosynthetic efficiency, oxidative stress markers, and ROS scavenging enzyme activities) and soil microbial communities (metabolic and structural diversity) will be studied.

## 2. Results

### 2.1. Effect of Biosynthesized AgNPs from Pseudomonas N5.12 on Cytoskeletal Structures in Arabidopsis Roots

**Figure 1:**
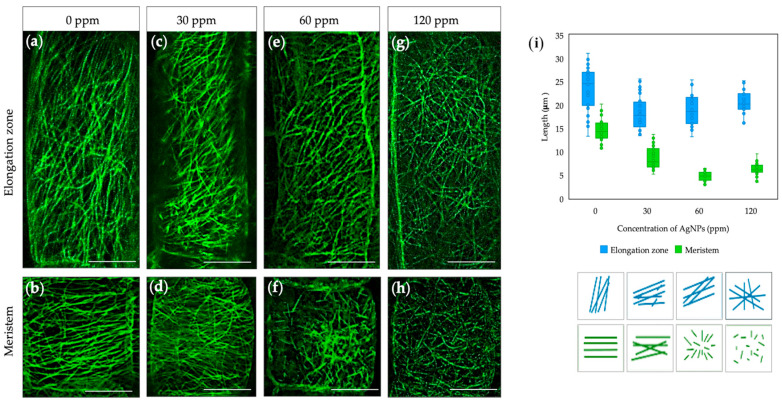
Organization of microtubules in *Arabidopsis gfp-map4* 1 h after exposure to different AgNP concentrations: 0 ppm (**a**,**b**); 30 ppm NPs (**c**,**d**); 60 ppm NPs (**e**,**f**); 120 ppm NPs (**g**,**h**). Average MT size in the cells of the elongation and meristematic zones (**i**). The images were taken sequentially with confocal microscopes from Leica Microsystems in the green channel for GFP detection (ex/em: 488/496–556 nm). Scale bars: 10 μm.

The physiological dose of AgNPs was evaluated by assessing the disruption of cytoskeleton structures, microtubules (MT), and actin filaments (AF). MT orientation varies across root zones, reflecting the different stages of cell growth and division. The arrangement of MT in the meristematic zone of native plants is perpendicular to the cell division plane (Figure 1a), while in the elongation zone, they reorganize to a longitudinal orientation to support cell elongation (Figure 1b). In NP-treated plants, the epidermal cells of the root elongation zone show MT reorientation 1-h after treatment with different concentrations of AgNPs. Specifically, after treatment with 30 ppm AgNP, the cells exhibit a predominantly longitudinal and oblique orientation (Figure 1c); at 60 ppm, most cells show an oblique arrangement (Figure 1e); and at 120 ppm, a chaotic MT arrangement predominates (Figure 1g). The stock solution (6000 ppm) caused a total disruption of the cytoskeleton (Supplementary Figure S1). NPs did not affect MT length in the elongation zone, which fluctuated within the values of control plants (from 18 to 23 μm) across all concentrations studied (Figure 1i, blue). Additional changes in MT organization were observed in the root meristematic cells. At a low concentration of AgNP (30 ppm), no significant changes in MT organization were detected, with most having a predominantly transverse orientation, as in the control (Figure 1d). Higher concentrations of NPs caused disruptions in the structure of MTs, resulting in short filaments randomly distributed throughout the cell cytoplasm (Figure 1f,h). In short, MT length was reduced by 57.7% and 68.4% following treatment with NPs at 60 and 120 ppm, respectively (Figure 1i, green). In summary, AgNP affects the orientation of MTs without dramatic changes in their organization, but higher concentrations shorten MTs.

**Figure 2:**
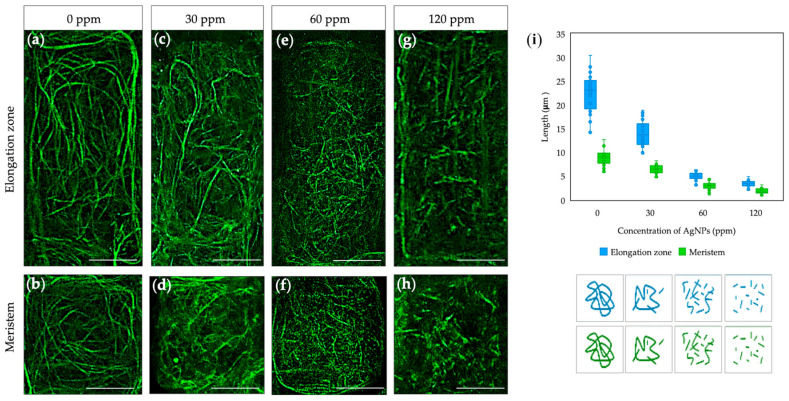
Effect of AgNP on the AFs of *Arabidopsis gfp-fabd2* cells 1 h after treatment: 0 ppm (**a**,**b**); 30 ppm NPs (**c**,**d**); 60 ppm NPs (**e**,**f**); 120 ppm NPs (**g**,**h**). Average AFs size in the cells of the elongation and meristematic zones (**i**). The images were taken sequentially with confocal microscopes from Leica Microsystems in the green channel for GFP detection (ex/em: 488/496–556 nm). Scale bars: 10 μm.

The effect of AgNPs on the structure of Actin Filaments (AFs) in *Arabidopsis* root cells was studied using *gfp-fabd2* mutants. The results revealed that AgNPs have a more pronounced impact on AF organization. Elongating cells contain a thin and highly dynamic network of AFs throughout the cytoplasm (Figure 2a). When seedlings are exposed to 30 ppm NPs, the AF network is thinned somewhat but is similar to that of the control (Figure 2c). At higher concentrations, AgNPs (60 and 120 ppm) cause AFs to break apart or fragment into shorter segments, disrupting the normal structure and organization of the AF network (Figure 2e,g). A similar pattern is observed in meristematic cells, where AgNPs cause the disintegration and depolymerization of AFs as the concentration increases from 30 to 120 ppm (Figure 2d,f,h).

As a result, AgNPs disrupt AF length and stability in different root cells. The average size of AFs decreases slightly following treatment with 30 ppm NPs; however, this change remained statistically non-significant, as it fluctuates within the range of the control values. In elongating cells, the size of AFs decreased by 77.6% and 84.8% following treatment with 60 ppm and 120 ppm NPs, respectively (Figure 2i, blue), indicating a significant disruption in the structure at higher concentrations. The same trend was detected in the cells of the meristematic zone, where the size of AFs decreased by 67.5% and 79.8% (with 60 ppm and 120 ppm NPs, respectively), indicating a concentration-dependent effect on actin structure (Figure 2i, green).

Therefore, low concentrations of AgNPs are less likely to cause significant toxicity or major disruption of cytoskeleton dynamics in *Arabidopsis* roots. Based on these data, a concentration of 30 ppm AgNPs was selected for further research on plant growth and development.

### 2.2. Biological Effects of AgNPs on Tomato Plants

**Figure 3:**
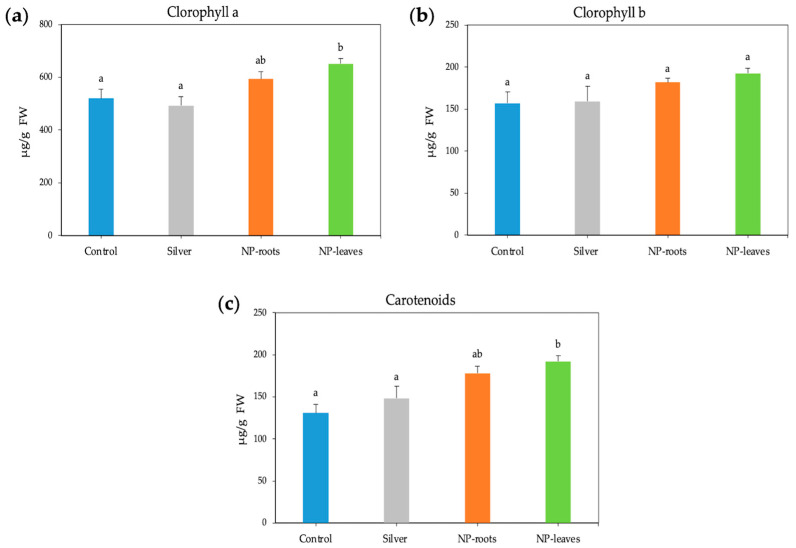
Photosynthetic pigments concentration (units) in 7-week-old tomato plants: chlorophyll *a* (**a**), chlorophyll *b* (**b**), and carotenoids (**c**). Treatments: Control—no treatment; NP-leaves—NP applied via foliar spray; NP-roots—NP applied via soil drench; Silver—treated with 1 mM AgNO_3_. Values are recorded as mean ± standard error of a triplicate experiment. Different letters represent significant differences determined by the ANOVA (LSD, *p* < 0.05).

Photosynthetic efficiency showed non-significant differences in any parameter, although non-photochemical quenching (NPQ) showed a notable non-significant increase (Supplementary Figure S2). NPs positively affected the levels of photosynthetic pigments, irrespective of the delivery method. Root treatment resulted in a 14.2% increase in chlorophyll *a* content, while the leaf treatment led to a significant increase of 25.2% (Figure 3a); however, chlorophyll *b* content showed non-significant variations compared to the control (Figure 3b). More notable changes were observed in carotenoid content, which increased by 36% and 46.6% following root and leaf treatments, respectively (Figure 3c). Silver nitrate did not affect the photosynthetic pigment concentration.

**Figure 4:**
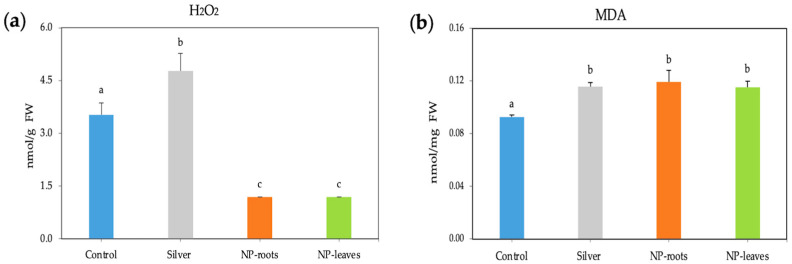
Oxidative stress markers (units): hydrogen peroxide (**a**) and malondialdehyde (**b**) in 7-week-old tomato plants. Treatments: Control—no treatment; NP-leaves—NP applied via foliar spray; NP-roots—NP applied via soil drench; Silver—treated with 1 mM AgNO_3_. Values are recorded as mean ± standard error of a triplicate experiment. Different letters represent significant differences determined by the ANOVA (LSD, *p* < 0.05).

**Figure 5:**
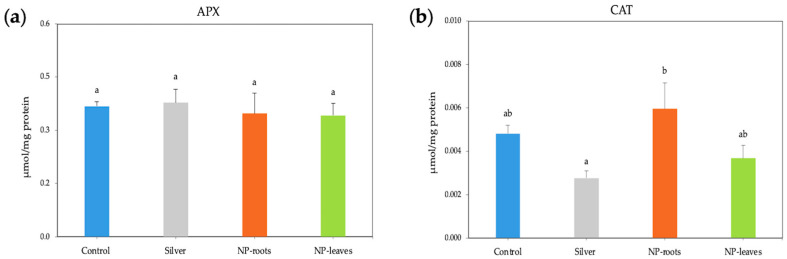
ROS Scavenging enzymes activity (units): ascorbate peroxidase (**a**) and catalase (**b**) in 7-week-old tomato plants. Treatments: Control—no treatment; NP-leaves—NP applied via foliar spray; NP-roots—NP applied via soil drench; Silver—treated with 1 mM AgNO_3_. Values are recorded as mean ± standard error of a triplicate experiment. Different letters represent significant differences determined by the ANOVA (LSD, *p* < 0.05).

**Figure 6:**
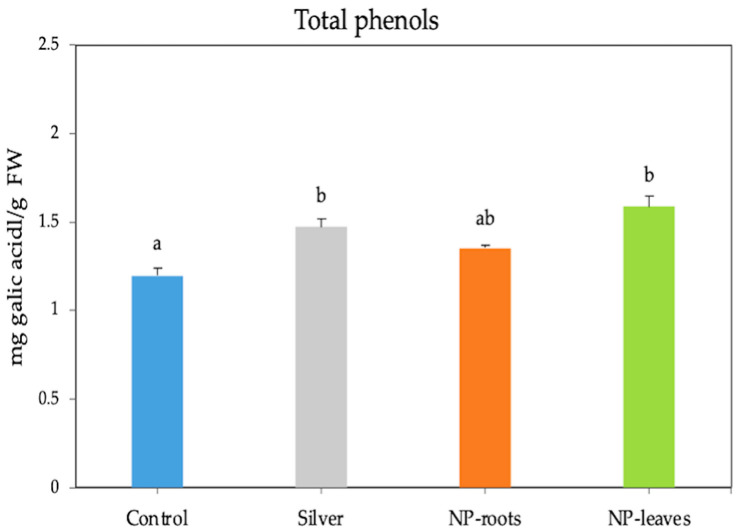
Total phenolic compounds (units) in 7-week-old tomato plants. Treatments: Control—no treatment; NP-leaves—NP applied via foliar spray; NP-roots—NP applied via soil drench; Silver—treated with 1 mM AgNO_3_. Values are recorded as mean ± standard error of a triplicate experiment. Different letters represent significant differences determined by the ANOVA (LSD, *p* < 0.05).

A significant decrease in the H_2_O_2_ content by almost three times was observed in both root- and leaf-treated plants. In contrast, when treated with 1 mM AgNO_3_, the H_2_O_2_ content increased by 1.3 times compared to the control (Figure 4a), and the MDA content was similarly enhanced by all treatments (Figure 4b). At the same time, the activities of the ROS scavenging enzymes APX and CAT under AgNP treatments were not statistically different from those of the control plants (Figure 5a,b). However, exposure to NPs significantly increased the total phenolic content in tomato plants, with the highest values observed in leaf-treated plants (1.588 mg/g FW) compared to the control and similar to silver nitrate (Figure 6).

### 2.3. Metabolic Activity of Soil Microbiota

**Figure 7:**
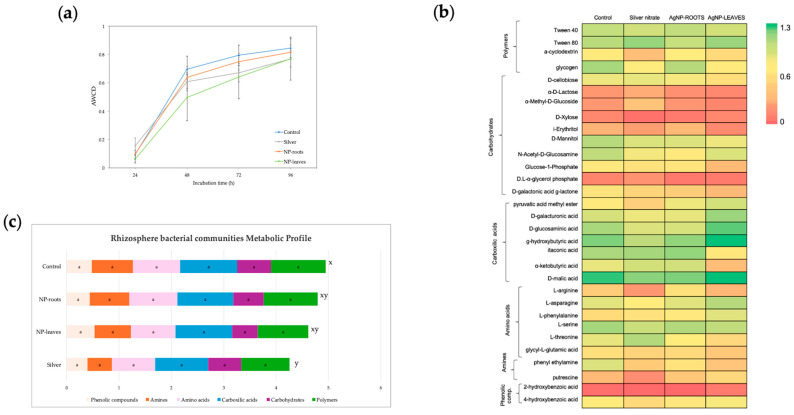
Metabolic activity of rhizosphere bacterial community. (**a**) Average well color development (AWCD) in Biolog Eco plate on 96 h incubation performed as the mean of the absorbance value of the 31 wells of Biolog Eco plate; (**b**) Heat map performed with the absorbance values of the 31 wells of Biolog Eco plate, measured at 96 h of incubation; (**c**) Metabolic profile of bacterial communities displayed as bars graph with the mean absorbance values of the substrates of each category (polymers, carbohydrates, carboxylic acids, amino acids, amines and phenolic compounds). Treatments: Control—no treatment; NP-leaves—NP applied via foliar spray; NP-roots—NP applied via soil drench; Silver—treated with 1 mM AgNO_3_. Different letters indicate statistically significant differences between treatments (x, y) or categories (a) as determined by the ANOVA (LSD, *p* < 0.05).

The average well-color development (AWCD) reflects the overall metabolic activity of the rhizosphere bacterial community. The kinetics of AWCD are shown in Figure 7a. To further investigate how AgNPs alter the metabolic profile of soil microorganisms, 31 carbon source utilization was analyzed. The heat map and metabolic profile obtained from the absorbance values of the different carbon sources of Biolog Eco plates are presented in Figure 7b and c, respectively. The metabolic fingerprint shown in the heat map was similar among all treatments, with AgNO_3_ treatment showing slight differences in the use intensity of a few wells compared with the control (α-cyclodextrin, L-arginine, L-threonine, Putrescine). The use intensity of polymers, carbohydrates, carboxylic acids, amino acids, amines, and phenolic compounds by rhizosphere bacteria was not altered by any of the treatments (Figure 7c). The metabolic profile was significantly different only between the AgNO_3_ and Control groups (Figure 7c). Metabolic diversity, calculated as the Shannon index, did not indicate significant differences between treatments (Control: 4.808 ± 0.023; Silver: 4.761 ± 0.017; NP-roots: 4.777 ± 0.019; NP-leaves: 4.722 ± 0.066).

### 2.4. Diversity of Microbial Communities

**Figure 8:**
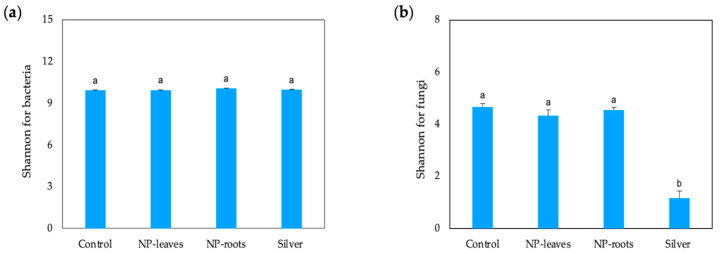
Shannon index for the diversity of bacterial (**a**) and fungal (**b**) communities under different treatments. Different letters represent significant differences determined by the ANOVA (LSD, *p* < 0.05). Treatments: Control—no treatment; NP-leaves—NP applied via foliar spray; NP-roots—NP applied via soil drench; Silver—treated with 1 mM AgNO_3_.

The structural diversity of rhizosphere microbial communities under different treatments was analyzed. Alpha biological diversity (Shannon-Weaver index) and abundance were calculated for both bacterial and fungal communities. The Shannon index for bacteria showed no significant changes between the treatments, suggesting that bacterial diversity remained stable under AgNP treatment (Figure 8a). The Shannon index for fungi was similar for the controls and both NP treatments; however, significantly lower values were found for the Ag treatment (Figure 8b). Beta diversity analysis revealed no significant differences (Supplementary Table S1).

**Figure 9:**
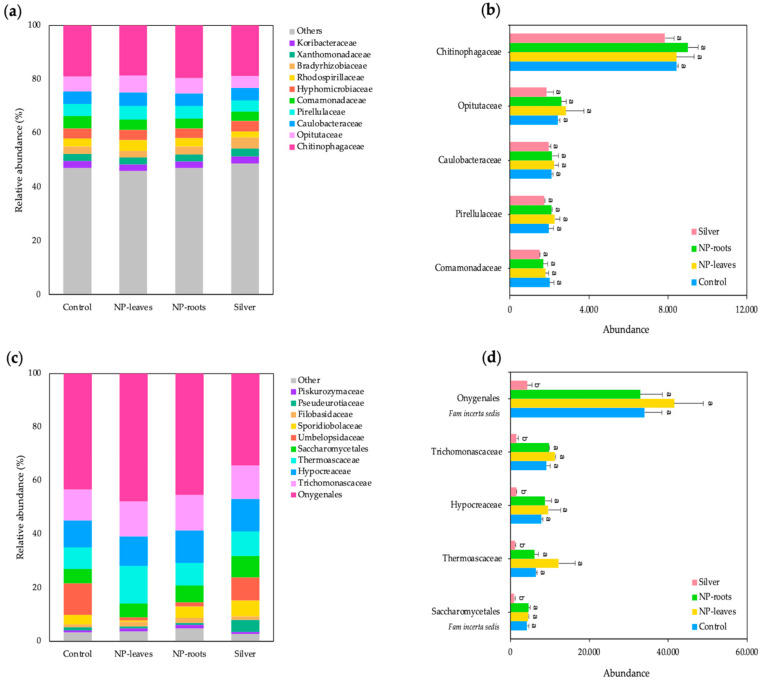
Relative abundance (%) of the top 10 bacterial families (**a**) and absolute abundance (number of OTUs) of the top 5 bacterial families. For each family, different letters indicate significant differences between treatments (**b**). Relative abundance (%) of the top 10 fungal families (**c**) and absolute abundance (number of OTUs) of the top 5 fungal families. For each family, different letters indicate significant differences between treatments (**d**). Different letters represent significant differences determined by the ANOVA (LSD, *p* < 0.05). Treatments: Control—no treatment; NP-leaves—NP applied via foliar spray; NP-roots—NP applied via soil drench; Silver—treated with 1 mM AgNO_3_.

The composition of the bacterial and fungal communities at the family level is presented in Figure 9. We identified the top 10 bacterial families across treatments, accounting for an average relative abundance of 53% of the total bacterial community, while the remaining 47% consisted of other less abundant families (Figure 9a). In particular, the family Chitinophagaceae was the most dominant, representing 19% of the total bacteria. No significant differences were observed in the abundance (number of OTUs) of the top five dominant bacterial families between the treatments (Figure 9b).

The fungal community predominantly consisted of 96% of the top 10 most abundant fungal families, with Onygenales (*Fam. incertae sedis*) accounting for 43% of the total fungal community (Figure 9c). Neither nanoparticle treatment (NP-leaves and NP-roots) significantly affected the abundance (number of OTUs) of the top five fungal families compared to the control, as shown in Figure 9. At the same time, Ag treatment (Silver) resulted in a significant decrease (LSD test, *p* < 0.05) in the absolute abundance of all studied families, with a decrease ranging from 76% to 88%, depending on the fungal family (Figure 9d).

## 3. Discussion

The present study provides preliminary evidence of the environmental safety of AgNPs in agriculture for AgNPs synthesized using metabolites from beneficial bacterial strains. This is a groundbreaking result, as the use of AgNPs in agriculture is on the rise, and evidence for environmental and health safety needs to be provided. Furthermore, the innovative approach in this study is grounded in the consideration of two potential mechanisms in rhizosphere communities: direct or systemic. First, the potential inhibitory effects of AgNPs on soil microbial communities due to AgNP antimicrobial activity [18] were ruled out by observations of microbial structure and activity. Secondly, approaching changes in microbial communities due to changes in exudate profiles induced by leaf-delivered NP rules out the potential negative effects driven by the plant.

The uniqueness of this study’s results relies on the bacterial strain used, which is able to trigger plant protection against pathogens and drought [20,21]; therefore, a similar or even better effect of the NP is foreseen. As a first step towards this goal, the present study was conceived to explore the potential negative effects on soil microbial communities or in plants in order to provide preliminary evidence of their ability to trigger plant metabolism and environmental safety. The results show that AgNPs can trigger plant metabolism without altering the structure and activity of microbial communities.

Determining biological activity requires sensitive and suitable markers. The cytoskeleton is an excellent marker for this goal since it reacts immediately to environmental stress, as both microtubules and microfilaments disassemble upon stress sensing to repolymerize within a short time if the perturbation is not toxic [14]. AgNPs can significantly impact the organization and function of MTs and AFs, as metal NPs may damage the plant’s cellular structures in the event of NP uncapping or Ag oxidation, as silver itself is ten times denser than the cytosol. The exact effects depend on various factors, including the size, shape, concentration, and surface properties of the nanoparticles, as well as the exposure duration [13]. Previous studies have shown that exposure to AgNPs leads to a significant reduction in the number and growth rate of MTs in *Arabidopsis* plants [14]. Some studies have shown that AgNPs might induce the bundling or aggregation of microtubules, which can alter the typical parallel arrangement of microtubules in plant cells [14,22]. In the case of AgNPs, the crown of bacterial metabolites stabilizes and caps the metal, preventing or eliminating toxicity, as evidenced by the non-significant changes in cytoskeleton arrangements at lower concentrations. Consistently, higher NP concentrations led to MT and AF shortening, suggesting physical interference when NP are too concentrated, as AgNPs can disrupt MTs and AFs organization (Figure 1 and Figure 2) by inhibiting polymerization and leading to depolymerization or the formation of unstable structures. This study highlights the concentration-dependent toxicity of AgNPs and their potential to interfere with crucial cellular processes such as cell division and elongation. Based on the cytoskeleton response, a physiologically active concentration was established in *Arabidopsis* using *gfp-map4* and *gfp-fabd2* mutants.

Nanoparticles with a size of less than 60 nm are particularly effective at entering plant tissues, as they can navigate plasmodesmata (20–50 nm) and stomata (10–60 μm) [23]. Their shape also influences their entry and transport efficiency, with spherical nanoparticles being the most effective nanoparticles. Spherical AgNPs typically demonstrate better penetration and distribution within plant tissues than irregularly shaped particles [24]. As the size of our AgNPs (average diameter of 20 nm) allows penetration through plant tissues, and tissues are similar in all plant species, we reasoned that the same concentration would be suitable for tomato plants. This statement is especially relevant for future applications in agriculture, so N5.12-AgNP can be used in different crops. Furthermore, as AgNPs are prone to systemic transport due to their size, two application systems were explored, considering the effects on soil rhizosphere communities from both ends, directly in the roots, or as a side effect of the plant’s metabolic changes, systemic transport within the plant, or by dripping from leaves.

A number of beneficial effects of biologically synthesized AgNPs, from seed germination to plant growth stimulation, have been reported [8,25]. Improving nutrient content, maintaining the structural integrity of chloroplasts, and other cellular components involved in photosynthesis or enhancing pigment synthesis in plants have also been described as mechanisms to improve plant fitness [26]. Although our AgNPs did not affect chlorophyll fluorescence, the total chlorophyll and carotenoid content significantly increased, consistent with other authors [27,28]. Interestingly, both treatments triggered tomato plants, with leaf treatments having a more pronounced effect than root treatments, indicating both a systemic effect from the roots and a direct effect when delivered to the leaves.

The oxidative stress response has been studied in line with the improvement of plant fitness. Reactive oxygen species (ROS) levels increase upon exposure to AgNPs, and ROS alteration is a general plant response to integrate environmental changes. ROS changes can have different effects depending on their concentration; while high ROS levels may damage tissues, low levels can trigger beneficial responses [29]. In response to stimuli, plant systems activate a mechanism for scavenging ROS by increasing the activity of antioxidant enzymes, as well as antioxidant phenols and flavonoids, to restore physiological homeostasis [7]. Our results are consistent with improving ROS homeostasis [29,30], as indicated by the significant decrease in H_2_O_2_ content; ROS control is based on antioxidant molecules like phenols instead of ROS-scavenging enzyme activities. In line with the lower H_2_O_2_ concentration indicative of non-stressing conditions, lower MDA levels were expected; however, an unexpected increase in MDA content was found in both root and leaf treatments, suggesting that MDA could be acting as a signaling molecule [31] in view of the improved physiological status of plants. The protective effects of bacterial metabolites on the NP crown are evidenced by the different responses shown by the increase in H₂O₂ levels compared to the control and treatment with 1 mM AgNO_3_.

The environmental safety of AgNPs was the second concern addressed in this work, as our AgNPs demonstrated *in vitro* antimicrobial activity against human and plant pathogens [18]. Microorganisms play a key role in soil fertility and are vital for supporting the functions of soil ecosystems. However, the presence of contaminants can interfere with these functions, including the cycling of nitrogen [32]. NPs participate in various physical, chemical, and biological processes, including vulcanization, flocculation, precipitation, and adsorption. These processes allow them to interact with soil organic matter, plants, and microorganisms [15]. NPs can impact rhizosphere microorganisms positively or negatively, depending on factors such as their size, concentration, soil composition, and microbial community in the rhizosphere [33]. Monitoring the overall metabolic activity of rhizosphere bacteria can provide insights into how AgNPs affect microbial ecosystems. In the literature, soil extract exposure (ex situ) to uncoated AgNPs and ionic silver resulted in a decrease in bacterial metabolic activity [34,35]. However, the non-toxic effects of N5.12-coated NP as either treatment had a minimal impact on the functionality of bacterial communities, probably due to N5.12 being a natural compound in the rhizosphere. However, when analyzing the metabolic profile (Figure 7c), a significant negative overall effect of AgNO_3_ was detected. This indicates that AgNP-functionalized N5.12 metabolites efficiently reduce silver to form NP and stabilize it, preventing the oxidation of Ag and, therefore, avoiding the potential negative impact of oxidized Ag on the metabolic activity of rhizobacteria, as revealed by the silver control. All this suggests that, at the concentrations used in our study, AgNPs coated with beneficial bacteria metabolites may have limited ecological consequences for soil microbial communities function.

Additionally, our results regarding the diversity of soil microbial communities indicate that the nanoparticle treatments (NP-leaves and NP-roots) do not cause significant changes in the composition of the top five bacterial and fungal communities (Figure 9). However, silver treatment (Silver) had a noticeable impact, significantly reducing the relative abundance of major fungal families. These findings support the notion that the form of silver (NPs versus ions) plays a crucial role in shaping microbial and fungal community composition, with AgNPs generally exerting a more stable and less toxic effect than their ionic counterparts [35]. This confirms the stabilization of the metal due to the corona of bacterial organic matter and its beneficial effects, as anticipated in our hypothesis based on the beneficial effect of the strain and its rhizosphere origin.

## 4. Materials and Methods

### 4.1. Biosynthesis of AgNP from Pseudomonas N5.12

AgNPs were obtained as described in Plokhovska et al. [18]. In summary, 24 h cell-free supernatants were mixed with 1 mM AgNO_3_ (2:4; *v*/*v*) and incubated at 37 °C for 24 h. After washing, the product was lyophilized and resuspended in 1 mL of milli Q water with a stock concentration of 6000 µg/mL. It was confirmed by UV-Vis spectra (430 nm) measured using a SPECTROstar Nano spectrometer (BMG LABTECH, Germany), and TEM images obtained with a Transmission Electron Microscope (JEOL JEM-1400Flash) showed spherical particles (20.71 ± 0.43 nm) with an organic crown of bacterial metabolites (Supplementary Figure S3).

### 4.2. Determination of Physiological Dose in Transgenic Arabidopsis

Five-day-old seedlings of *Arabidopsis thaliana* plants (GFP-MAP4 and GFP-FABD2) with fluorescently labeled microtubules and actin filaments, respectively, were used for the experiments. The mutant lines were obtained from the European Arabidopsis Stock Centre (NASC, UK; https://arabidopsis.info, accessed on 13 July 2023) under stock codes N799990 and N73159. *Arabidopsis* seeds were sterilized in 70% EtOH (1.5 min) and 10% sodium hypochlorite (10 min) and exposed to cold stratification for 2 days. After stratification, seeds were sown on agar plates under sterile conditions. Seedlings were grown in a vertical position in a culture chamber Pol-Eko KK 500 SMART PRO (Pol-Eko, Poland) at 22 °C and 70% relative humidity (RH) with a 12 h light period. Five-day-old seedlings were transferred for 1 h to different concentrations of AgNPs (30, 60, and 120 ppm) to study their effect on the plant cytoskeleton. Changes in the organization of MTs and AFs in the cells of the elongation zone and root meristem were observed using a Leica Stellaris 8 Confocal Microscope (SAI-MICROCON; Leica Microsystems, Germany), https://www.uspceu.com/en/research/research-support/servicio/microscopia-confocal (accessed on 13 July 2023). The fluorescent signal from GFP was excited at 488 nm and emitted at 505/530 nm wavelength. To measure the lengths of MTs and AFs, ImageJ software (Version 1.8.0_345) was used. Measurements were performed on confocal microscope images, specifically within the elongation zone and meristematic cells (n = 30).

### 4.3. Experimental Design: Effect of AgNPs on Tomato Plants and Microbial Communities

Experiments were conducted to investigate whether the physiological dose of AgNPs in *Arabidopsis* was effective in tomato plants *Solanum lycopersicum* F1-Hybrid (Razymo RZ, The Netherlands), and to explore changes in rhizosphere microbial communities. Two methods of AgNP delivery were evaluated: leaf spray and root drench. Therefore, the experimental treatments under evaluation are NP delivered by soil drench (NP-root) and NP delivered by leaf spray (NP-leaves); a non-inoculated control is run in parallel, as well as silver-treated plants (silver) to prove that the results are due to NP and not silver.

**Figure 10:**
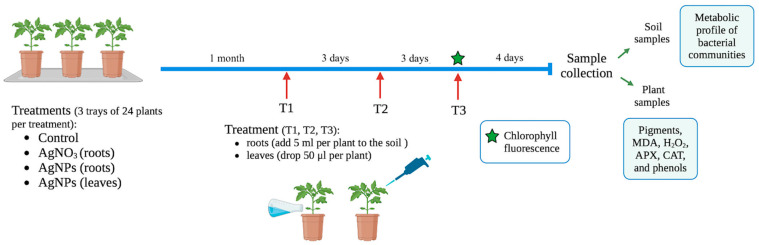
Experimental design.

The experimental design is shown in Figure 9. Seeds were sown in trays (4) with 12 alveoli. Each tray was watered with 1 L of tap water every two days and grown in a greenhouse under a natural photoperiod with a day-night temperature range of 10 °C to 30 °C. One month after sowing, 24 seedlings (two trays) were inoculated with AgNPs, 12 seedlings (one tray) were treated with 1 mM AgNO_3_ (silver), and 12 seedlings (1 tray) were used as controls. AgNPs (30 ppm) were delivered to the leaves (NP leaves) by adding a drop to the newest leaf (50 μL/plant; one tray) or by soil drenching (NP root) (5 mL/plant; one tray). Treatments were delivered 3 times, with 3 days between each application; the first application was performed on 4-week-old tomato plants (Figure 10). Four days after the last treatment, the plant shoots were harvested, powdered in liquid nitrogen, and stored at −80 °C. Rhizosphere soil was sampled and stored at −80 °C until further analysis. Twelve plants per treatment were sampled, and material from 4 plants was pooled and constituted a replicate.

### 4.4. Effects on Plant Physiology

#### 4.4.1. Chlorophyll Fluorescence

Photosynthetic efficiency was assessed based on chlorophyll fluorescence emitted by photosystem II. Chlorophyll fluorescence was measured using a Pulse-Amplitude Modulated (PAM) fluorometer HansatechFM2 (Hansatech Instruments Ltd., UK) on 1 h dark-adapted leaves. Variable fluorescence (Fv) was calculated as the difference between the maximum fluorescence (Fm) and minimum fluorescence (Fo). The maximum photosynthetic efficiency of photosystem II (PSII) was calculated as the ratio of Fv/Fm. Next, the leaves were continuously irradiated with red-blue actinic beams and equilibrated to record the steady-state fluorescence signal (Fs). The effective PSII (φPSII) and non-photochemical quenching coefficient (NPQ) were calculated as follows: φPSII = (Fm′ − Fs)/Fm′ and NPQ = (Fm − Fm′)/Fm′. Five plants per treatment were used to measure all parameters [20].

#### 4.4.2. Photosynthetic Pigments

One hundred milligrams of leaf powder was dissolved in acetone 80% (1 mL) (*v*/*v*), and after overnight incubation at 4 °C, samples were centrifuged at 10,000 rpm for 5 min in a Hermle Z233 M-2 centrifuge, and the absorbance was measured at 647, 663, and 470 nm to quantify chlorophylls (*a*, *b*) and carotenoids on a spectrophotometer Biomate 5. Concentrations of chlorophylls *a* and *b* was calculated as in Porra et al. [36] and carotenoids as proposed by Lichtenthaler [37].

Chl a (μg/g FW) = [(12.25 × Abs663) − (2.55 × Abs647)] × V(mL)/weight (g).

Chl b (μg/g FW) = [(20.31 × Abs647) − (4.91×Abs663)] × V(mL)/weight (g).

Carotenoids (μg/g FW) = [(1000 × Abs470) − (1.82×Chl a) − (85.02 × Chl b)/198] × V(mL)/weight (mg).

#### 4.4.3. Oxidative Stress Markers

H_2_O_2_ was quantified as described by Shukla et al. [38]. Two hundred milligram samples were vortexed with 2.0 mL of 0.1% (*w*/*v*) trichloroacetic acid (TCA) in an ice bath and then centrifuged for 20 min at 10,000× *g*. Five hundred mL of the supernatant were mixed with the same volume of 10 mM of potassium phosphate buffer (pH 7.0) and 1 mL of potassium iodide solution (1 M). The absorbance was measured spectrophotometrically at 390 nm after 5 min. The amount of H_2_O_2_ formed was estimated from a standard curve and expressed as nmol/g H_2_O_2_ of FW.

Malondialdehyde (MDA) content was determined using the method described by Hu et al. [39]. Briefly, 2 mL of 10% (*v*/*v*) trichloroacetic acid (TCA) was added to 0.1 g of powder and centrifuged for 30 min at 20,000× *g* at 4 °C. The supernatant (1 mL) was mixed with 4 mL of 20% with thiobarbituric acid (TBA) 0.5% (*v*/*v*). The supernatant (1 mL) was mixed with 4 mL of 20% TCA and 0.5% TBA. The reaction mixture was incubated for 30 min at 95 °C and then cooled to room temperature. After centrifugation at 10,000× *g* for 10 min, the absorbance of the supernatant at 532 and 600 nm was measured. Finally, the concentration of MDA (nmol/g FW) was determined using the following equation: [(Abs 532 − Abs 600)]/(ε × FW), where ε is the molar extinction coefficient (155 mM^−1^/cm).

#### 4.4.4. Enzyme Activities

To determine enzyme activity, a buffered enzyme extract was prepared (ten mg of powder in 1 mL of 0.1 M pH 7 potassium phosphate buffer supplemented with 2 mM Phenylmethyl sulfonyl fluoride) and manipulated at 4 °C. After sonication (10 min) and centrifugation (10 min) at 14,000 rpm, the soluble proteins were determined in the supernatant. Fifty μL of supernatant was mixed with 250 μL of Bradford reagent to determine the total protein on ELISA 96-well plates (Thermo Fisher Scientific, USA). After an incubation period of 30 min at room temperature, the absorbance at 595 nm was determined using a Full-Automatic Microplate Reader MB-580 (Heales Medical Equipment, China). A calibration curve was constructed using commercial BSA (0.05 and 2 mg/mL) to interpolate the absorbance and determine the protein concentration. Enzyme activities related to ROS scavenging were determined in the supernatant using a spectrophotometer.

Ascorbate peroxidase (APX) was measured as described by García-Limones et al. [40]. One hundred microliters of enzyme extract were added to 860 μL of potassium phosphate buffer (50 mM pH 7.0), followed by 120 μL of 2.5 mM sodium ascorbate. The reaction was initiated by adding 120 μL of 50 mM H_2_O_2_. The oxidation of ascorbate was detected by a decrease in absorbance at 290 nm. The activity was calculated using an extinction coefficient of 2.8 mM^−1^ cm^−1^.

Catalase (CAT) was determined as described by García-Limones et al. [40]. Fifty microliters of the enzyme extract were mixed with 980 μL of 50 mM pH 7.0 potassium phosphate buffer and 120 μL of 200 mM H_2_O_2_. Absorbance at 240 nm was measured immediately, and after 20 s; a decrease in absorbance indicates H_2_O_2_ breakdown. The activity was calculated using an extinction coefficient of 36 mM^−1^/cm.

#### 4.4.5. Total Phenols

The total phenol content was quantified using Folin–Ciocalteu reagent (Sigma Aldrich, St. Louis, MO, USA) with modifications [41]. Twenty microliters of extract were mixed with 250 μL of Folin-Ciocalteu agent, 750 μL of a 20% solution of Na_2_CO_3,_ and 950 μL H_2_O. After 2 h at room temperature, the absorbance was measured at 760 nm. A gallic acid calibration curve was constructed (r = 0.99). The results are expressed in mg of gallic acid equivalents per 100 g of FW.

### 4.5. Effects on Microbial Communities

#### 4.5.1. Metabolic Profile of Soil Microbiota

Biolog^®^ ECO plates (BIOLOG Inc., Hayward, CA, USA) were used to assess the metabolic profile and diversity 8 of bacterial communities [42]. Biolog ECO plates are 96-well microplates that contain 31 substrates in triplicate (providing three repetitions per replicate, three replicates; n = 9) and three control wells without substrates.

A bacterial suspension was prepared by homogenizing 2 g of rhizosphere soil in 20 mL of sterile distilled water by stirring for 15 min. The homogenate was centrifuged for 10 min at 2500 rpm, and the supernatant was filtered through glass wool. The bacterial suspension was resuspended in MgSO_4_ 10 mM to achieve 95% transmittance at 620 nm. The wells of the plate were inoculated with 150 μL of the bacterial suspension. Plates were incubated at 25 °C in the dark, and the absorbance at 595 nm of the wells was measured every 24 h until 96 h using a Full-Automatic Microplate Reader MB-580 (Heales Medical Equipment, China). Each absorbance value was corrected by subtracting the blank (corrected absorbance) from it.

The corrected mean absorbance of all wells, known as the average well color development (AWCD), was calculated and plotted against the incubation time to obtain the growth curves of the microbial community in the wells of the plate. In these curves, the incubation time selected was when the growth of microorganisms reached the stationary phase, namely, 96 h. The metabolic diversity of each sample was calculated using the Shannon-Weaver diversity index calculated with the corrected absorbance values of the selected incubation time (96 h) [43]:

H = −S [ni/N × Log ni/N],

where ni is the corrected absorbance of each substrate (well) and N = Sum of the 31 corrected absorbance values.

#### 4.5.2. Metagenomic Analyses of the Rhizosphere Bacterial and Fungi Communities

Metagenomic analyses were performed by AllGenetics & Biology SL (Spain; www.allgenetics.eu, accessed on 11 June 2024). DNA was extracted from 250 mg of each rhizosphere sample using the DNeasy PowerSoil Pro kit (Qiagen, Germany) following the manufacturer’s instructions, with an extraction blank included to check for contamination. The DNA was resuspended in 50 μL and quantified using a Qubit High Sensitivity dsDNA Assay (Thermo Fisher Scientific, USA).

For the rhizosphere bacterial community library preparation, the 16S rRNA gene (domains V4-V5, around 400 bp) was amplified using the following primers: forward 515F-Y (5′-GTGYCAGCMGCCGCGGTAA-3′) [44] and reverse 926R (5′-CCGYCAATTYMTTTRAGTTT-3′) [45].

For rhizosphere fungal community library preparation, a fragment of the ITS1 genomic region (of around 300 bp) was amplified using the following primers: Forward ITS1F (5′ CTTGGTCATTTAGAGGAAGTAA 3′) [46] and reverse ITS2 (5′ GCTGCGTTCTTCATCGATGC 3′) [47]. In both libraries, the pool was sequenced in a fraction (1/16) of a MiSeq PE300 flow cell.

EasyMAP, a user-friendly online platform for evaluating 16S rRNA sequencing data, was used [48]. For alpha diversity analysis, EasyMAP produces “observed OTUs”, “Faith’s Phylogenetic Diversity”, “Shannon index”, and “Pielou’s evenness”. LEfSe was used to identify microbes with significant differences across groups, and EasyMAP was used to provide taxonomic differential abundance analysis. Upon running a Wilcoxon test between distinct subgroups, the default alpha value was 0.05, and the standard threshold for the absolute value of the logarithmic linear discriminant analysis (LDA) score was two. The default alpha value for the Kruskal–Wallis test between the statistical groups was 0.05. Finally, to predict the function of the microbes, the Greengenes database (version 13_8, 99% OTU) and VSEARCH [49] were used for close-reference clustering, ensuring that representative sequences were mapped to the Greengenes database. PICRUSt locates the matching function in the KEGG database using a mapped Greengenes ID.

For fungi, taxonomic assignment of each ASV (Amplicon Sequence Variant) was performed using a pre-trained classifier of the UNITE reference database [50], updated in May 2021. Specifically, the feature-classifier approach classify-sklearn implemented in QIIME 2 [51], with the confidence parameter set at 0.80, was used.

### 4.6. Statistics

To evaluate the existence of the statistical differences in the physiological parameters evaluated, the Shannon-Weaver index, and the absolute abundance (number of OTUs) of the top five bacterial and fungal families between treatments), a one-way ANOVA with repetitions was performed. IBM SPSS Statistics (Version 29.0.2.0) was used to verify the homoscedasticity and normality of the variance prior to ANOVA, ensuring that all analytical conditions were met. The LSD test was employed when significant differences (*p* < 0.05) were observed.

## 5. Conclusions

In summary, the following conclusions can be drawn:

The metabolites from *Pseudomonas* N5.12 formulated in AgNPs have biological activity that is dose-dependent, as shown by cytoskeleton alterations.

Physiologically active doses are low and effective in various plant species.

N5.12-AgNPs contribute to oxidative stress homeostasis in plant cells by modulating antioxidant molecules for ROS scavenging without involving major changes in specific antioxidant enzymes.

N5.12-AgNPs did not significantly affect the overall microbial diversity or activity, indicating environmental safety.

N5.12-AgNPs enhance plant fitness while maintaining rhizosphere microbial balance.

## Data Availability

The datasets generated and/or analyzed during the current study are available in the Zenodo repository: https://doi.org/10.5281/zenodo.14626361 (accessed on 25 April 2025).

## Supplementary Materials

The following supporting information can be downloaded at: https://www.mdpi.com/article/10.3390/plants14111655/s1, Figure S1: Organization of microtubules in *Arabidopsis gfp-map4* after exposure AgNP stock solution; Figure S2: The effect of AgNPs on photosynthetic efficiency in tomato plants; Figure S3: Characteristics of biosynthesized AgNPs from *Pseudomonas* sp. N5.12. Table S1. Dissimilarity test by PERMANOVA among different treatments.

## Abbreviations

The following abbreviations are used in this manuscript:

AgNP	Silver nanoparticles
MT	Microtubules
AF	Actin Filaments
APX	Ascorbate peroxidase
CAT	Catalase
MDA	Malondialdehyde
H_2_O_2_	Hydrogen peroxide
AWCD	Average Well Color Development
