# Supplementary material for: Pseudomonas sp. N5.12 Metabolites Formulated in AgNPs Enhance Plant Fitness and Metabolism Without Altering Soil Microbial Communities
Source: Plants (Basel). 2025 May 29;14(11):1655. doi: 10.3390/plants14111655 (PMC12157261; doi:10.3390/plants14111655)
Supplement: Supplementary file 1 [file plants-14-01655-s001.zip › plants-3637912-supplementary.pdf]

Supplementary materials

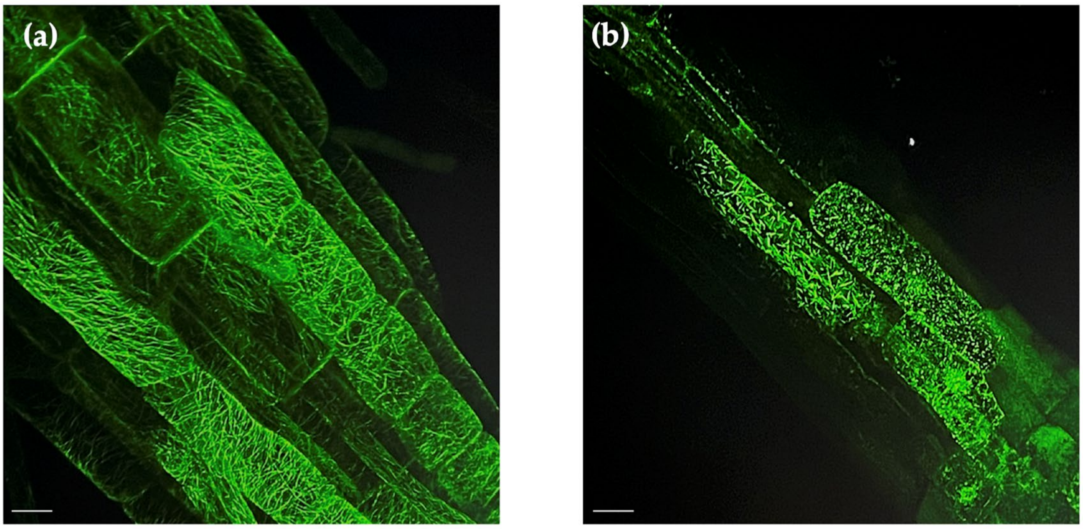

Figure S1. Organization of microtubules in Arabidopsis *gfp-map4* after exposure AgNP stock solution: (**a**) control; (**b**) 6000 ppm. The images were taken sequentially with confocal microscopes from Leica Microsystems in the green channel for GFP detection (ex/em: 488/496–556 nm). Scale bars: 10 μm.

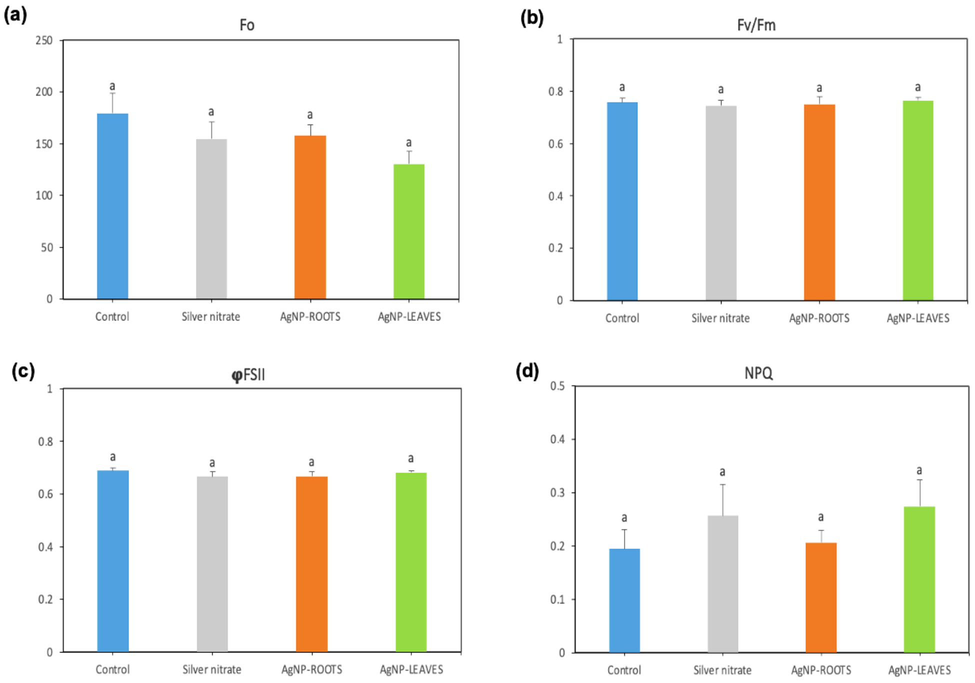

Figure S2. The effect of AgNPs on photosynthetic efficiency in tomato plants: (**a**) minimal fluorescence (Fo); (**b**) maximum photosynthetic efficiency (Fv/Fm); (**c**) effective PSII quantum yield (φPSII); (**d**) non-photochemical quenching (NPQ). Values are recorded as mean ± standard error of a triplicate experiment. No statistical difference was found according to the ANOVA test ($p < 0.05$).

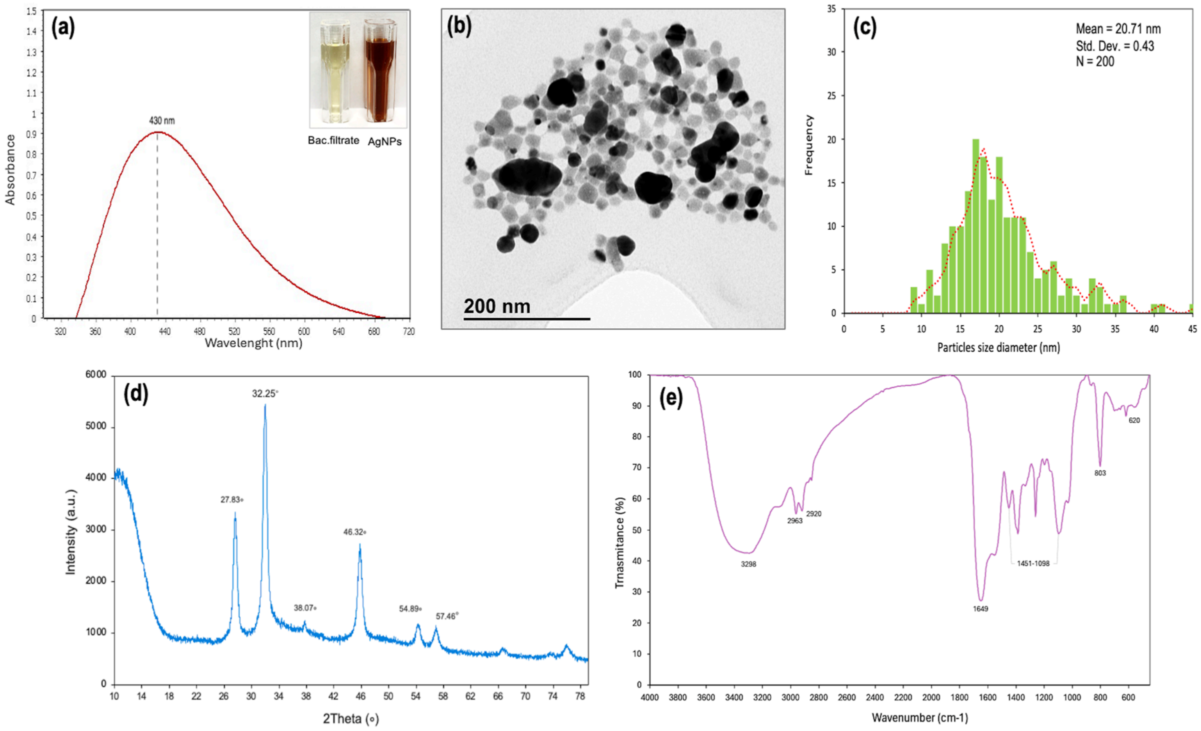

Figure S3. Characteristics of biosynthesized AgNPs from *Pseudomonas* sp. N5.12: (**a**) UV-Vis absorption spectra; (**b**) TEM image; (**c**) histogram of the particle size distribution; (**d**) XRD analysis; (**e**) FTIR spectra.

Table S1. Dissimilarity test by PERMANOVA among different treatments.

*$p < 0.05$

	Treatments	*p*-value
Bacteria	Control / NP-leaves	1.0
	Control / NP-roots	0.66
	Control / Silver	0.325
	NP-leaves / NP-roots	0.659
	NP-leaves / Silver	0.317
	NP-roots/Silver	0.351
Fungi	Control / NP-leaves	0.349
	Control / NP-roots	0.335
	Control / Silver	0.352
	NP-leaves / NP-roots	0.67
	NP-leaves / Silver	0.327
	NP-roots/Silver	0.335
